# Prevalence of *Cytauxzoon felis* infection in healthy cats from enzootic areas in Arkansas, Missouri, and Oklahoma

**DOI:** 10.1186/s13071-014-0618-z

**Published:** 2015-01-08

**Authors:** Theresa E Rizzi, Mason V Reichard, Leah A Cohn, Adam J Birkenheuer, Jared D Taylor, James H Meinkoth

**Affiliations:** Department of Veterinary Pathobiology, Oklahoma State University, Stillwater, OK USA; Department of Veterinary Medicine and Surgery, University of Missouri, Columbia, MO USA; College of Veterinary Medicine, North Carolina State University, Raleigh, NC USA

**Keywords:** Arkansas, Cats, *Cytauxzoon felis*, Missouri, Oklahoma

## Abstract

**Background:**

Infection with *Cytauxzoon felis* in domestic cats can cause fever, lethargy, depression, inappetence, icterus, and often death. With a high mortality rate, cytauxzoonosis was historically considered a fatal disease. Within the last 15 years, cats with or without treatment have been recognized as chronically infected survivors of *C. felis* infection. Our objective was to determine the prevalence of *C. felis* in healthy domestic cats from Arkansas, Missouri, and Oklahoma.

**Methods:**

Infection with *C. felis* was determined using DNA extracted from anticoagulated whole blood and PCR amplification using *C. felis*-specific primers. Chi-square, Fisher’s exact tests, and odds ratios were used to compare proportions of cats infected with *C. felis*.

**Results:**

Blood samples were collected from 902 healthy domestic cats between October 2008 and April 2012. DNA from *Cytauxzoon felis* was detected in 56 of 902 (6.2%; 95% confidence interval, 4.7–7.9) samples. The highest prevalence of *C. felis* infection (15.5%; 10.3–21.7) was observed in cats from Arkansas, followed by cats from Missouri (12.9%; 6.1–24.0), and cats from Oklahoma (3.4%; 2.2–5.1). Cats sampled in Arkansas and Missouri were 5.1 and 4.2, respectively, times more likely to be chronically infected with *C. felis* than cats from Oklahoma.

**Conclusions:**

Infection with *C. felis* is common in domestic cats through Arkansas, Missouri, and Oklahoma. The high prevalence of *C. felis* reported herein suggests that infected domestic cats are likely reservoirs of infection for naive felines. The high prevalence of *C. felis* substantiates the importance for the use of approved acaricides on cats to prevent cytauxzoonosis.

## Background

*Cytauxzoon felis* is a tick-transmitted protozoan parasite that can cause fatal disease in domestic cats and some wild captive felids [1-5]. Cytauxzoonosis was first described in 1976 [6]. Historically, bobcats (*Lynx rufus*) have been considered reservoirs for *C. felis* and domestic cats (*Felis catus*) as aberrant dead-end hosts [7-10]. More recently, a study demonstrated transmission of *C. felis* from chronically infected domestic cats to naive cats via tick bite, demonstrating cats are competent reservoirs for *C. felis* [11,12].

Experimental transmission of *C. felis* has been demonstrated with *Dermacentor variabilis* [8,11] and *Amblyomma americanum* [11,12]. The occurrence of cytauxzoonosis coincides with the distribution and seasonal activity of *A. americanum* [11], possibly explaining why cytauxzoonosis is not present in domestic cats in regions where *C. felis* is present in bobcats but *A. americanum* are not found [9,15]. Cytauxzoonosis is enzootic in the south-central United States, but cases have been identified in states extending to the mid-Atlantic coast [16-18].

Onset of disease typically follows 10–14 days after *C. felis*-infected ticks feed on naive cats. Cats become depressed, anorectic, markedly pyretic, dehydrated, pale, and icteric as schizont-laden macrophages occlude vasculature. Most cats die within a week of initial illness. Treatments for cytauxzoonosis have been tried with limited success [19,21,22]. A recent study demonstrated a 60% survival in cats given the combination of atovaquone and azithromycin plus supportive care [22], historically the disease was believed to be nearly always fatal with few reported survivors [19,20,23].

In addition to cats that survive illness and can remain chronically infected, chronically infected cats have been recognized with no known antecedent illness [23-25]. Chronically infected cats have been reported in north-western Arkansas and north-eastern Oklahoma [23,24], Florida and Tennessee [25], and Arkansas and Georgia [26]. We hypothesized that chronically infected cats might serve as an additional reservoir of *C. felis* for naive domestic cats. Because domestic cats are more likely to live near other domestic cats than near bobcats, these reservoir cats might assume an important role in disease transmission. The purpose of the current study was to determine the prevalence of *C. felis* infection in domestic cats in an enzootic area with high incidence of disease.

## Methods

**Figure 1:**
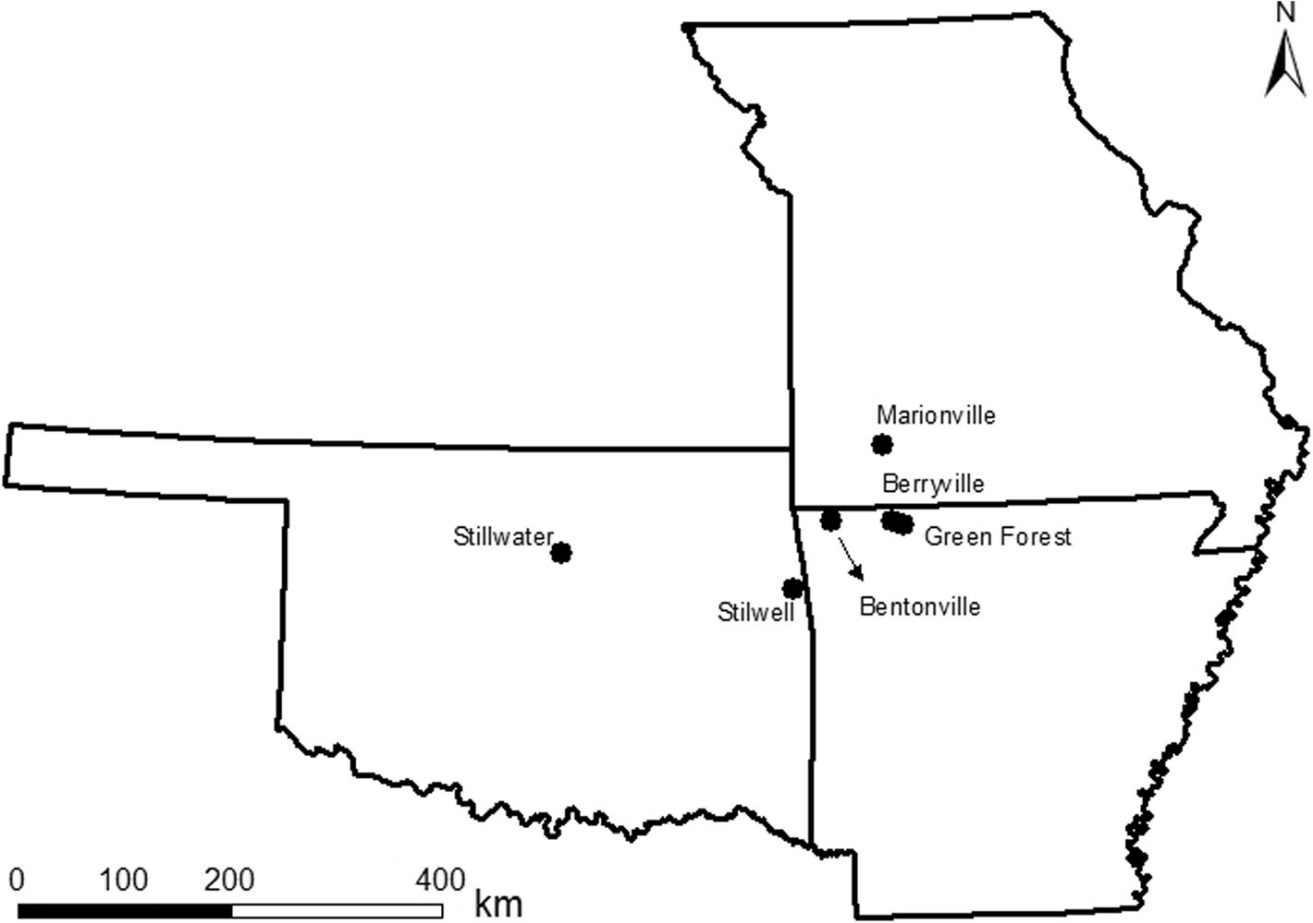
**Locations of participating veterinary clinics.** Veterinary clinics in Arkansas, Missouri, and Oklahoma that submitted feline blood samples tested for infection with Cytauxzoon felis.

Participation in this survey was solicited from veterinarians in Oklahoma, Missouri and Arkansas (Figure 1). Whole blood samples collected in EDTA from domestic cats for routine procedures or illness unrelated to cytauxzoonosis at private veterinary clinics, animal shelter/spay/neuter programs, or client cared for feral cats in Oklahoma, Missouri and Arkansas were submitted for this study from October 2008 through April 2012. The samples were used for other blood testing prior to submission for this study. Criteria for inclusion were domestic cats at least 6 months of age that were not exhibiting signs of illness consistent with *C. felis* infection. Cats previously diagnosed with C. felis infections were excluded from the study. All blood samples submitted were stored at 4°C up to 6 months until shipment to North Carolina State University for testing.

Blood samples were analyzed for *C. felis* infection using previously described methods [25]. Briefly, DNA was extracted from whole blood using the QIAmp DNA Blood Mini Kit or Magattract DNA Blood Mini M48 Kit (Qiagen Inc., Valencia, CA). Amplification of a portion of the 18S rRNA gene of *C. felis* was accomplished using PCR and primers specific to *C. felis*. Primer sequences were: 5’-GCGAATCGCATTGCTTTATGCT-3’and 5’-CCAAATGATACTCCGGAAAGAG-3’. Positive controls consisted of DNA extracted from known *C. felis*-infected cat whole blood and negative controls consisted of purified (no DNA) water. Samples that were negative for *C. felis* infection were screened for the presence of PCR inhibitors via amplification of a glyceraldehyde 3-phosphate dehydrogenase (GAPDH) pseudogene as previously described [27]. Sample processing, DNA extraction, master mix assembly, PCR amplification, and post amplification processing were performed in separate areas to avoid amplicon contamination. Good laboratory procedures were employed to ensure uniformity, consistency, reliability, and reproducibility of results.

The prevalence of *C. felis* infection in cats was calculated according to Bush et al. [28]; 95% confidence intervals were calculated according to Sterne’s exact method [29] using Quantitative Parasitology 3.0 [30]. Proportions of cats infected with *C. felis* were compared with Chi-square and Fisher’s exact tests using Sigma Plot 12.5 (Systat Software Inc., San Jose, CA). Odd ratios [31] were calculated to express differences in the proportion of cats infected from Arkansas, Missouri, and Oklahoma.

## Results

A total of 902 feline blood samples were tested from 2008–2012:161 from AR, 62 from MO, and 679 from OK (Table 1). Of these 902 samples, *C. felis* was detected in 56 cats, resulting in an overall prevalence (95% confidence interval) of 6.2% (4.7–7.9). Of the 161 cats collected in AR, *C. felis* was detected in 25, resulting in a prevalence of 15.3% (10.3–21.7). Of the 62 cats collected in MO, *C. felis* was detected in 8, resulting in a prevalence of 12.9% (6.1–24.0). Of the 679 cats collected in OK, *C. felis* was detected in 23, resulting in a prevalence of 3.4% (2.2–5.1). Statistically discernable differences (*X*^2^ = 37.458, df = 2, P = <0.0001) were detected in the proportion of cats infected with *C. felis* in AR, MO, and OK. The prevalence of *C. felis* infected cats in AR (15.3%) and MO (12.9%) were statistically higher (*X*^2^ = 32.730, df = 1, P = <0.0001; *X*^2^ = 10.570, df = 1, P = 0.001, respectively) than that of cats collected from OK (3.4%). Cats sampled in AR and MO were 5.1 and 4.2, respectively, times more likely to be infected with *C. felis* than cats from OK.

**Table 1:** **Prevalence of** ***Cytauxzoon felis*** **in cats collected in Arkansas, Missouri, and Oklahoma from 2008–2012**

**State**	**Sample origin**	**Number of samples**	**Number of cats infected**	**Prevalence (95% CI)**
Arkansas	Clinic A (Bentonville)	8	1	12.5% (0.6–50.0)
	Clinic B (Bentonville)	63	2	3.2% (0.5–10.8)
	Clinic C (Berryville)	30	4	13.3% (4.7–29.8)
	Clinic D (Green Forest)	60	18	30.0% (19.6–42.9)
Missouri	Clinic E (Marionville, MO)	62	8	12.9% (6.1–24.0)
Oklahoma	Clinic F (Stillwater & surrounding areas)	349	7	2.0% (1.0–4.1)
	Clinic G (Stillwell)	77	13	16.9% (9.7–27.2)
	Clinic H (Stillwater)	253	3	1.2% (0.3–3.5)
Total		902	56	6.2% (4.7–7.9)

Four community veterinary practices (clinics A, B, C, and D) in AR contributed samples with most obtained from client-owned cats (Table 1). All four of these clinics are located within approximately a 60 miles radius of each other in the north-western part of AR. All four clinics reported treating many cats with both fatal and non-fatal cytauxzoonosis throughout the years. Blood samples of 71 cats were submitted from clinics A and B in Bentonville, AR with 2 cats infected with *C. felis*. Thirty samples were submitted from clinic C in Berryville, AR with 4 cats testing positive (13.3%). Clinic D, located in Green Forest, AR (approximately 10 miles east of Berryville) submitted 60 samples with 18 testing positive (30.0%). The prevalence of *C. felis* in cats from Arkansas clinics were statistically discernible (*X*^2^ = 18.030, df = 3, P = 0.0004) from each other. Multiple comparisons of the proportion of cats infected with *C. felis* demonstrated the prevalence of *C. felis* in cats from Clinic D (30%) was statistically higher (*X*^2^ = 14.331, df = 2, P = <0.001) than those from Clinic B (3.2%).

Sixty-two cat samples from Marionville, MO all came from a single, client-managed outdoor population (Table 1). The client reported deaths attributed to cytauxzoonosis within this population of cats in the past. Eight samples tested positive by PCR (12.9%).

A total of 679 blood samples were submitted from veterinary practices and animal shelters located in central and eastern OK (Table 1). Of the 679 samples, 23 were positive by PCR for *C. felis* (3.4%). The prevalence of *C. felis* in cats from Oklahoma clinics were statistically discernible (*X*^2^ = 48.737, df = 2, P = <0.0001) from each other. Multiple comparisons of the proportion of cats infected with *C. felis* demonstrated the prevalence of *C. felis* in cats from Clinic G (16.9%) was statistically higher (*X*^2^ = 27.969, df = 1, P = <0.001; *X*^2^ = 28.348, df = 1, P = <0.001 than those of Clinics F (2.0%) and H (1.2%), respectively. Thirteen of the 77(16.9%) blood samples from Stillwell, OK (eastern OK approximately seven miles from the Arkansas border) were infected with *C. felis.* The majority of samples collected in OK were from the Stillwater area (central OK). Prior to the current study, relatively few cats subclinically infected with *C. felis* were known from central OK.

## Discussion

This is the first study to document the prevalence of chronic *C. felis* infection in domestic cats in a region of the United States with a high incidence of clinical cytauxzoonosis. The prevalence of *C. felis* (6.2%) in the current study is higher than the previously reported 0.3% in free roaming cats in Florida, North Carolina and Tennessee [25]. In addition, there were overall differences in the prevalence of *C. felis* in cats among Arkansas, Missouri, and Oklahoma with statistically discernible differences in the prevalence of *C. felis* in cats from AR and OK.

Cytauxzoonosis is common in this area of the country from spring to early fall [32,33]. There have been reports of domestic cats either recovering from clinical infection or being parasitemic without evidence of clinical disease [8,16,20,21,23-25,35,36,38]. Meinkoth *et a*l. [24] identified 18 domestic cats that survived natural infection with *C. felis* in eastern OK (Catoosa and Tahlequah) and north-west AR (Berryville, Fayetteville, Clarksville). They concluded treatment was not a likely explanation for the cats’ survival because some cats had no treatment, and others were treated with therapies not effective against *C. felis*. Possible explanations for survival include strain variation in virulence, innate immunologic response, or variations in tick inoculum. Strain variation is unlikely to be the entire explanation as many of the healthy, chronically infected cats were from households and neighborhoods where lethal cytauxzoonosis was also reported.

The prevalence of *C. felis* in apparently healthy cats from limited geographic areas suggests that there may be less virulent strains of *C. felis*. In 2009, *C. felis* isolates from blood samples obtained from 88 domestic cats in Georgia and Arkansas were analyzed. An association between infections with *C. felis* containing particular internal spacer regions (ITS) and survival rates of infected cats was noted in addition to genotype variability from samples originating in AR and GA [34]. However, in a subsequent study the ITS regions in asymptomatic cats were found to be the same as ITS regions previously detected in mostly fatal infections. The investigators concluded that analyzing ITS regions, while suitable for studying geographic variability, was not useful in determining pathogenicity of the strains [26]. Additional research will be required to identify genetic markers reflective of the virulence of *C. felis* strains.

It has been postulated cats surviving cytauxzoonosis could serve as reservoirs of infection for competent vectors [11,24,35], as cats remain parasitemic for extended periods [12,26,36]. The relative significance of such a reservoir would depend on the number of chronically infected cats, which historically was assumed to be low. The results of this study would support the contention that these cats could represent a substantial reservoir. The widespread occurrence of cats subclinically infected with *C. felis* in disparate areas suggests that chronically infected cats are present and available as reservoirs of infection across geographic regions.

Reichard *et al.* demonstrated that virulent infection could be transmitted from a chronically infected domestic cat to another cat via *A. americanum* vector [11]. A survivor of natural infection with *C. felis* was used to acquisition feed *A. americanum* nymphs. The *C. felis*-infected nymphs were allowed to molt to adults, which successfully transmitted *C. felis* to a naive cat. The recipient cat experienced clinical disease, but survived infection. In a subsequent transmission trial [12], similar cat-to-cat transmission was confirmed when *A. americanum* fed on the donor cat from the previous transmission trial were able to induce clinical cytauxzoonosis in four cats [13]. These transmission trials not only confirmed *A. americanum* as a tick vector for *C. felis*, but that cats with subclinical disease could be reservoirs of infection for naive domestic cats.

There have been reports of cytauxzoonosis survivor cats resistant to challenge with virulent *C. felis* [19,20], unpublished observation]. It is possible that immune response to initial infection of *C. felis* in chronically infected cats is sufficient to protect against subsequent challenge. Further research is necessary to determine the possibility of developing biologics that offer passive immunity to cats with clinical cytauxzoonosis or protection via immunization [36].

Cats with subclinical cytauxzoonosis may be persistently parasitemic for prolonged periods [37]. Since inclusion in this study, additional follow-up has become available for some cats, with several remaining parasitemic for years. In one cat undergoing treatment for intestinal lymphoma, the number of erythrocytes containing piroplasms as seen on blood film analysis increased after initiation of chemotherapy, suggesting immunosuppression may exacerbate parasitemia. To the authors’ knowledge, no cats recrudesced with cytauxzoonosis since recognition of subclinical infection.

With the realization that domestic cats are competent reservoirs of *C. felis* infection, information regarding the prevalence of carriers becomes increasingly important. Cats at highest risk of being infected with *C. felis* include those whose home ranges are in low density residential areas, along urban edges, with access to wooded cover, and in close proximity to natural or unmanaged habitat, all of which may lead to closer proximity to ticks that have fed on bobcats [32]. However, domestic cat reservoirs for the pathogen may not only be more likely to live in close proximity to other domestic cats than would bobcats, but they may also be moved during owner relocation to geographic areas where *C.felis* has not been endemic. Any region with *A. americanum* or *D. variabilis* could potentially allow for transmission of pathogen from these carrier cats. Because the geographic range of *A. americanum* has dramatically expanded in recent years [38], this could potentially lead to infection in areas where it has not been previously seen.

The high prevalence of *C. felis* in enzootic areas emphasizes the need to use measures to prevent tick exposure both to carrier cats that might act as reservoirs, and to naive cats that might be exposed to infection. Unfortunately, acaricides for use on cats are limited. Flumethrin, a synthetic pyrethroid safe to use on cats, has demonstrated efficacy for preventing the transmission of *C. felis* by *A. americanum* [39] and is labelled to repel and kill *D. variabilis*. Keeping cats indoors, combined with regular use of flumethrin, offers the best protection both against infection of naive cats, and transmission of *C. felis* from chronically infected carriers.

## Conclusions

In recent years domestic cats with or without treatment have been recognized as chronically infected survivors of *C. felis* infection. Our study demonstrates a prevalence of *C. felis* infection in domestic cats of 6.2% in an area of the United States with a high incidence of clinical cytauxzoonosis. Since recent studies have demonstrated domestic cats are competent reservoirs for *C. felis* and are more likely to live in close proximity to other domestic cats than would bobcats, the prevalence of infected cats will likely increase. Additionally, because of owner relocation, *C. felis* may be introduced in geographic areas with *A. americanum* or *D. variabilis* where *C.felis* is not endemic. Cytauxzoonosis is often diagnosed by observing piroplasms within erythrocytes during blood film evaluation. Since cats with subclinical disease can remain parasitemic for prolonged periods, chronic carriers presenting for any febrile illness may be mistakenly diagnosed with acute cytauxzoonosis. Because PCR would also be positive in these cats, it cannot be used to distinguish between carrier and acute infection. Prevention remains paramount by keeping cats indoors combined with regular use of flumethrin.
